# Morphological and Mechanical Tube Feet Plasticity among Populations of Sea Urchin (*Strongylocentrotus purpuratus*)

**DOI:** 10.1093/iob/obae022

**Published:** 2024-07-08

**Authors:** C A Narvaez, A Y Stark, M P Russell

**Affiliations:** Department of Biology, Rhode Island College, 600 Mt Pleasant Ave., Providence, RI 02908, USA; Department of Biology, Villanova University, 800 E. Lancaster Ave., Villanova, PA 19085, USA; Department of Biology, Villanova University, 800 E. Lancaster Ave., Villanova, PA 19085, USA; Department of Biology, Villanova University, 800 E. Lancaster Ave., Villanova, PA 19085, USA

## Abstract

Sea urchins rely on an adhesive secreted by their tube feet to cope with the hydrodynamic forces of dislodgement common in nearshore, high wave-energy environments. Tube feet adhere strongly to the substrate and detach voluntarily for locomotion. In the purple sea urchin, *Strongylocentrotus purpuratus*, adhesive performance depends on both the type of substrate and the population of origin, where some substrates and populations are more adhesive than others. To explore the source of this variation, we evaluated tube foot morphology (disc surface area) and mechanical properties (maximum disc tenacity and stem breaking force) of populations native to substrates with different lithologies: sandstone, mudstone, and granite. We found differences among populations, where sea urchins native to mudstone substrates had higher disc surface area and maximum disc tenacity than sea urchins native to sandstone substrates. In a lab-based reciprocal transplant experiment, we attempted to induce a plastic response in tube foot morphology. We placed sea urchins on nonnative substrates (i.e., mudstone sea urchins were placed on sandstone and vice versa), while keeping a subgroup of both populations on their original substrates as a control. Instead of a reciprocal morphological response, we found that all treatments, including the control, reduced their disc area in laboratory conditions. The results of this study show differences in morphology and mechanical properties among populations, which explains population differences in adhesive performance. Additionally, this work highlights the importance of considering the impact of phenotypic plasticity in response to captivity when interpreting the results of laboratory studies.

## Resumen

Los erizos de mar utilizan una secreción adhesiva en sus pies ambulacrales para adherirse al sustrato y resistir fuerzas hidrodinámicas. Los pies ambulacrales se adhieren fuertemente, pero pueden despegarse voluntariamente para moverse. En el erizo morado, *Strongylocentrotus purpuratus*, el desempeño del sistema de adhesión depende del tipo de sustrato y la población de origen donde algunas poblaciones y sustratos tienen mejor desempeño adhesivo que otros. Para explicar el origen de esta variación, evaluamos la morfología (área del disco) del pie ambulacral y las propiedades mecánicas (tenacidad máxima del disco y fuerza necesaria para romper el tubo que conecta el disco con el animal) de poblaciones que se encuentran adheridas a sustratos con diferentes litologías: arenisca, lodolitas, y granito. Encontramos diferencias entre las poblaciones donde erizos de la población que vive en lodolitas tienen tenacidad del disco más alta y discos más grandes que erizos que viven en arenisca. En un experimento de laboratorio, intentamos inducir una respuesta plástica en la morfología del pie ambulacral. Para esto, pusimos erizos en sustratos diferentes a los que normalmente se adhieren (erizos que en encuentran en lodolita los pusimos en arenisca y viceversa) y dejamos erizos en su sustrato original como control. Sin embargo, en lugar de una respuesta plástica en la morfología del pie ambulacral, encontramos que todos los tratamientos, incluido los controles, redujeron el área del disco en condiciones de laboratorio. Los resultados de este estudio muestran diferencias en morfología y propiedades mecánicas entre las poblaciones, lo que explica diferencias en el desempeño del sistema de adhesión. Además, este estudio demuestra la importancia de considerar el impacto de plasticidad fenotípica al momento de interpretar estudios de laboratorio.

## Introduction

Sea urchins play a critical role as consumers, bioeroders, and prey species in shallow benthic communities like kelp forests, sea grass beds, and coral reef ecosystems (Steneck 2013). These mobile herbivores can be found in wave-swept intertidal and shallow subtidal habitats, which are among the most stressful environments on earth, largely because of extremely variable and often intense hydrodynamic forces (Denny 1988). Therefore, in the dynamic intertidal zone, maintaining secure attachment, either through mechanical interlocking (i.e., interlocking of spines with rocky substrates) or chemical adhesion (via tube feet), under fluctuating environmental conditions is critical for sea urchin survival. Indeed, displacement from benthic substrates for nonswimming organisms may result in mortality or displacement to less desirable habitats (e.g., Carrington et al. 2009).

To avoid displacement, sea urchins use temporary chemical adhesion mediated by tube feet—external extensions of the water vascular system (Hyman 1955). Tube foot morphology varies depending on their functional specialization (Flammang 1996). Disc-ending tube feet are used for feeding (i.e., catching and moving food to the mouth from all locations on the body), maintaining position, and locomoting across various substrata (Fenner 1973). The disc is viscoelastic (i.e., deforms elastically under rapidly applied forces and behaves viscously under slowly acting forces [Vincent 2012]) and has a duo-glandular adhesive system that contains two types of secretory cells (i.e., adhesive and de-adhesive), allowing for strong but reversible adhesion while grazing along rocky substrates (Santos et al. 2009). Adhesive cells secrete a thin adhesive, bonding the tube foot to the substrate; while de-adhesive cells use secretions to de-bond the disc (Hermans 1983; Flammang and Jangoux 1993; Flammang 1996). The tube foot stem is a fluid-filled cylinder connecting the disc to the body that extends and contracts from changes in the volume of celomic fluid (Flammang 1996; Santos et al. 2009). The stem bears the load applied to sea urchins by external forces, but when subjected to a constant pull, the stem can suffer material failure before the disc is detached from the substratum, leading to tube foot amputation (Santos and Flammang 2007; Narvaez et al. 2020, 2022).

Sea urchin tube feet morphology and mechanical properties can vary intra- (i.e., body location) and interspecifically (i.e., species). For instance, tube feet morphology and mechanical properties vary depending on the position along the oral–aboral body axis (i.e., oral: bottom of the organism—facing the substrate; ambital middle of the organism halfway between oral and aboral; aboral: top of the organism—facing the water column) (Fenner 1973; Flammang 1996; Leddy and Johnson 2000). In *Strongylocentrotus droebachiensis*, tube feet located on the oral side require more tensile force to be broken (measured as breaking force) than any other location and have thicker stem walls to presumably enhance attachment. Tube feet located in the ambitus have a lower average breaking force than those on the oral side, thin stem walls, and are longer than oral and aboral tube feet, which could allow for their use in both respiration and attachment. Finally, tube feet located in the aboral side have the lowest breaking force of all locations, thin stem walls, and are suited for respiration (Leddy and Johnson 2000). In contrast, *Holopneustes purpurascens* tube feet morphology and mechanical properties are similar along all body locations (Connolly et al. 2017).

Sea urchins also exhibit remarkable plasticity. Over short timescales, sea urchins can change their morphology (e.g., test allometry and thickness [Selden et al. 2009; Russell et al. 2018]), physiology (e.g., reproductive output [Russell 1998]), and behavior (e.g., foraging and fleeing behavior [Harding and Scheibling 2015]) in response to the environment. Tube feet are no exception; previous research showed that sea urchin adhesion exhibits phenotypic plasticity in response to substrate characteristics (i.e., where they make adhesive contact), hydrodynamic forces, and water temperature fluctuations (Santos and Flammang 2007; Cohen-Rengifo et al. 2018, 2019; Stark et al. 2020). In *Paracentrotus lividus*, whole animal tenacity (whole animal detachment force divided by total attachment area, expressed in MPa) increases with water temperature (Santos and Flammang 2007) and whole animal attachment force (force required to remove an organism from a substrate [N]) is positively correlated with maximum wave height experienced a few days before sampling (Santos and Flammang 2007). At a smaller scale, disc detachment force increases when attaching to rougher substrates (Santos et al. 2005), and stem extensibility (dimensionless measure of length change at breaking point) and toughness (energy required to extend and break the stem scaled area [MJ m^−3^]) are higher in sea urchins found in sites more exposed to waves (Cohen-Rengifo et al. 2017). Even adhesive protein expression in tube feet discs is plastic and decreases when sea urchins are removed from the field and placed in an aquarium (Toubarro et al. 2016).

In the Eastern Pacific (CA, USA) three intertidal populations of the purple sea urchin, *Strongylocentrotus purpuratu*s, show population-level plasticity in whole animal attachment force (Stark et al. 2020). These populations inhabit and adhere to rock substrates of different hardness, roughness, and friability: mudstone, granite, and sandstone (Russell et al. 2018). Interestingly, independent of the substrate tested, sea urchins from the population native to mudstone (Palomarin Beach) and granite (Bodega Bay) had higher whole animal attachment force than sea urchins native to sandstone (Bean Hollow Beach) (Stark et al. 2020). These results suggest that populations native to mudstone and granite: 1) increase attachment force by increasing attachment area, which can be achieved by either attaching more tube feet (behavioral choice) or having larger discs (morphological plasticity), or both, 2) have higher disc tenacity, and/or 3) have stems capable of resisting higher loads. While there are no studies assessing the adhesive behavior, disc or stem mechanical properties in these populations, Narvaez et al. (2020) measured disc surface area among two of these populations and found that sea urchins native to mudstone (Palomarin Beach) have larger discs than sea urchins native to sandstone (Bean Hollow Beach). This morphological difference suggests that sea urchins in these populations exhibit adhesion-related plasticity that could be related to native substrate characteristics. In this study, we explore substrate lithology as a potential driver of the differential adhesive performance found among sea urchins from these three populations by assessing tube foot morphology (disc surface area) and mechanical properties (maximum disc tenacity [maximum disc detachment force divided by disc area] and stem breaking force).

To increase surface area for attachment and help prevent dislodgment, the three focal populations are found inside self-carved rock pits of different sizes: Bodega Bay has small and shallow pits, while Palomarin Beach and Bean Hollow Beach have large and deep pits (Russell et al. 2018). Hence, sea urchins found in deeper pits should be able to use both oral and ambital tube feet for attachment, while urchins found in shallow pits should use mostly oral tube feet. In this study, we examine whether there is functional specialization in tube feet adhesive performance among body locations (oral, ambital, and aboral).

Our study has two components: a field mensurative study (Hurlbert 1984) and a laboratory-based reciprocal transplant experiment. The goal of the field mensurative study was to assess tube foot morphology (disc surface area) and mechanical properties (stem breaking force, maximum disc tenacity) among populations of *S. purpuratus* inhabiting pits in sites with different substrate lithology (Palomarin Beach: native to mudstone, Bodega Bay: native to granite, and Bean Hollow Beach: native to sandstone) and among different body locations (oral, ambital, and aboral). Our predictions were: First, sea urchins from populations native to mudstone (Palomarin Beach) and granite (Bodega Bay) would have larger disc surface areas than those native to sandstone (Bean Hollow Beach), as shown by previous studies (Narvaez et al. 2020). Second, maximum disc tenacity would be comparable among populations, as we do not expect disc viscoelastic behavior or adhesive secretion to be influenced by the native substrate when tested on glass (see the “Methods” section). Third, stem breaking force would be comparable among populations, as this part of the tube foot is not in contact with the substrate and should not be driven by substrate-related plasticity. Fourth, for populations inhabiting deep pits (Palomarin Beach and Bean Hollow Beach), tube feet on the oral and ambital body location will have higher adhesive performance than those found on the aboral body location, as sea urchins in deep pits can use oral and ambital tube feet for adhesion to the substrata. For the population inhabiting shallow pits (Bodega Bay) however, tube feet located on the oral body will have higher adhesive performance than those found on the ambital and aboral body location, as sea urchins in shallow pits should use mostly oral tube feet for adhesion to the substrata. Finally, tube feet on the aboral body location will have similar adhesive performance among the three populations, as these tube feet should not be influenced by substrate lithology because they are not in contact with the substrate.

After the field mensurative study, we conducted a laboratory-based reciprocal transplant experiment aimed to test the hypothesis that tube feet morphological differences among the populations were environmentally induced plastic responses due to differences in substrate lithology. In this experiment, sea urchins were confined to a native (control) or nonnative substrate for 109 days and tube feet morphological and mechanical properties were measured before and after exposure to the substrate treatments. We hypothesized that oral tube foot discs of sea urchins native to rougher and larger grain size substrate (i.e., sandstone) would increase in surface area after attaching to smoother substrates (and vice versa), and that stem and disc mechanical properties would not be affected by substrate.

## Methods

We assessed sea urchin tube foot morphological (disc surface area) and mechanical properties (maximum disc tenacity, stem breaking force) among populations native to three sites in California (USA) with different rock types: Granite—Bodega Bay (38°19ʹ08.28ʺN, 123°04ʹ27.85ʺW), Mudstone—Palomarin Beach (37°55ʹ48.81ʺN, 122°44ʹ44.09ʺW), and Sandstone—Bean Hollow Beach (37°13ʹ36.08ʺN, 122°24ʹ41.70ʺW) (Russell et al. 2018). The sedimentary mudstone and sandstone rocks are less dense and more friable (easier to erode) than the metamorphic granite rock. However, mudstone is composed of fine mud/slit particles (<0.06 mm in diameter) making its surface smoother and more similar to granite than sandstone. Sandstone is more friable and has a larger particle size (1.00–0.25 mm in diameter) than the other two substrates (Russell et al. 2018).

Between June 12 and 18, 2018, sea urchins (*n* = 21–22 per site, see below) were carefully removed from rock pits, minimizing the loss of tube feet. Sea urchins of a wide size range (35–75 mm in diameter) were selected from each population, but Bodega Bay sea urchins were smaller (35–50 mm in diameter), likely because of mass mortality events in the area in previous years (Russell et al. 2018). Sea urchins were collected from pits that have different depth and size: pits in the soft mudstone and sandstone substrates are deep and large; while pits in the hard granite substrate are shallow and small (Russell et al. 2018). These differences are related to the ability of the sea urchin to create pits by consuming and eroding the substrate and results in different allometries among sea urchins from these populations: sea urchins from mudstone and sandstone pits have higher height to diameter ratios than sea urchins found in granite pits (Russell et al. 2018). Thus, we used test volume (mL) instead of diameter as a metric for size. To account for allometric differences among populations, we estimated test volume using a formula for an oblate spheroid (Ebert 1988; Elliott et al. 2012):


\begin{eqnarray*}
{\mathrm{Volume}} &=& \frac{4}{3}\pi \times {{\left( {0.5 \times {\mathrm{Diameter}}} \right)}^{2 }}\times ( {0.5\ \times {\mathrm{\ Height}}}),
\end{eqnarray*}

where volume is expressed in mL and diameter and height are measured in cm. Henceforth, all measurements reported as averages will represent the mean ± 1 SE.

Nondestructive disc surface area and maximum disc tenacity measurements were assessed using one set of sea urchins (Palomarin Beach: *n* = 12, volume = 43.12 ± 5.49 mL; Bean Hollow Beach: *n* = 12, volume = 39.72 ± 3.43 mL; and Bodega Bay, *n* = 11, volume = 21.15 ± 1.24 mL) and stem breaking force was measured using a different set of sea urchins (*n* = 10 for all populations—volume = Palomarin Beach: 32.20 ± 5.88 mL; Bean Hollow Beach: 22.18 ± 4.10 mL; and Bodega Bay: 22.18 ± 1.21 mL) to reduce the handling time and additional stress associated with the destructive sampling required to break the stem (see below).

### Mensurative field study

Sea urchins were transported to University of California, Davis, Coastal and Marine Science Institute's Bodega Bay Marine Laboratory (BML, Bodega Bay, USA), and measurements of test volume, tube foot morphology (disc surface area), and mechanical properties (maximum disc tenacity, stem breaking force) were conducted, within 18 h of collection, at three body locations: oral, ambital, and aboral.

All the procedures involved placing a single sea urchin in a “sponge restraint”—a concave sponge that exposed the target body location and covered the nontargeted tube feet. The soft concave sponge was placed around the nontargeted body locations to prevent tube feet damage and attachment. The sponge, tightly secured around the sea urchin, was then fitted into a PVC pipe (13 cm in diameter) glued (with hot-melt adhesive or “hot glue”) to either the bottom (for disc surface area and stem breaking force measurements) or side (maximum disc tenacity measurements) of a clear 4.7-L plastic bucket (18 × 18 × 18 cm) filled with seawater. When submerged, sea urchins extended tube feet of the uncovered body location allowing measurements of disc surface area, disc attachment force (needed to calculate maximum disc tenacity), and stem breaking force (see diagram of the setup in Fig. S1; Narvaez et al. 2020, 2022).

#### Disc surface area

Disc surface area (mm^2^) was measured by photographing tube feet discs attached to the transparent glass. To capture photos, sea urchins were placed inside the sponge restraint, leaving the target body location exposed, and a large glass petri dish was placed on top (with a 1-mm scale). A picture was taken with an Olympus Tough TG-6 digital camera (12MP, Olympus Corporation, Tokyo, Japan) after at least 10 discs were visibly attached to the glass (see fig. 1 in Narvaez et al. 2020 for a photograph of the setup). Surface area was measured by delineating the disc using the “oval” tool feature of the Image J software program (Abramoff et al. 2004). We calculated the mean disc surface area of 10 discs per body location of each sea urchin to determine the average disc surface area of each individual and body location. The mean disc surface area (mm^2^) per sea urchin and body location was then divided by the sea urchin volume (mL) to obtain the scaled disc surface area (mm^2^ mL^−1^).

#### Maximum disc tenacity

Maximum tube foot disc tenacity (MPa) was calculated as the maximum adhesive force (N) required to detach one tube foot from a standard substratum (glass) divided by the average disc surface area of that individual in m^2^. We measured maximum adhesive force by allowing a single tube foot to adhere to the side of a glass capillary tube (1.5 mm outside diameter) connected by surgical silk to a hand-held 5 N digital force gauge (FGE-XY, Nidec-Shimpo Instruments, Glendale Height, IL, USA). Once attached, we manually pulled upward at a consistent rate (approximately 2.32 cm/s), until the disc detached from the capillary tube (diagram of the setup in Fig. S1A). To standardize this procedure, the same person conducted all trials (C.A.N.). This procedure was repeated five times on different tube feet per body location, and the highest value of each body location was used to estimate the tube foot maximum disc tenacity of each individual and body location. We used the highest value instead of the average because we were interested in comparing the highest performance value an organism can produce (i.e., their performance maximum).

#### Stem breaking force

Tube foot stem breaking force (N) was measured as the maximum force required to cause material failure when pulling vertically (upward) at a constant rate. The stem breaking force was measured by clamping a metal clip (brand BlastCase Steel Toothless Alligator Clips; John Miller, Inc.) to an extended tube foot. The metal clip had squares of sandpaper glued to the tips to increase the surface area, which prevented breakage when clamping and kept the tube foot from slipping during the pull. The metal clip was connected to a hand-held 5N digital force gauge (FGE-XY, Nidec-Shimpo Instruments, Glendale Heights, IL, USA) with monofilament fishing line (4-lb Shakespeare Omniflex, Columbia, SC, USA). We clamped the clip to the extended tube foot at approximately half its length and manually pulled upward, at a consistent rate (approximately 2.54 cm/s), until the stem broke (diagram of the setup in Fig. S1B, Narvaez et al 2022). To standardize this procedure, the same person conducted all trials (A.Y.S). The maximum force required to break a tube foot was recorded. We calculated the stem breaking force of five stems per body location of each sea urchin to determine the average stem breaking force of that individual and body location. The average stem breaking force (N) per sea urchin and body location was then divided by the sea urchin volume to obtain the scaled stem breaking force (N mL^−1^).

### Laboratory-based reciprocal transplant experiment

**Fig. 1:**
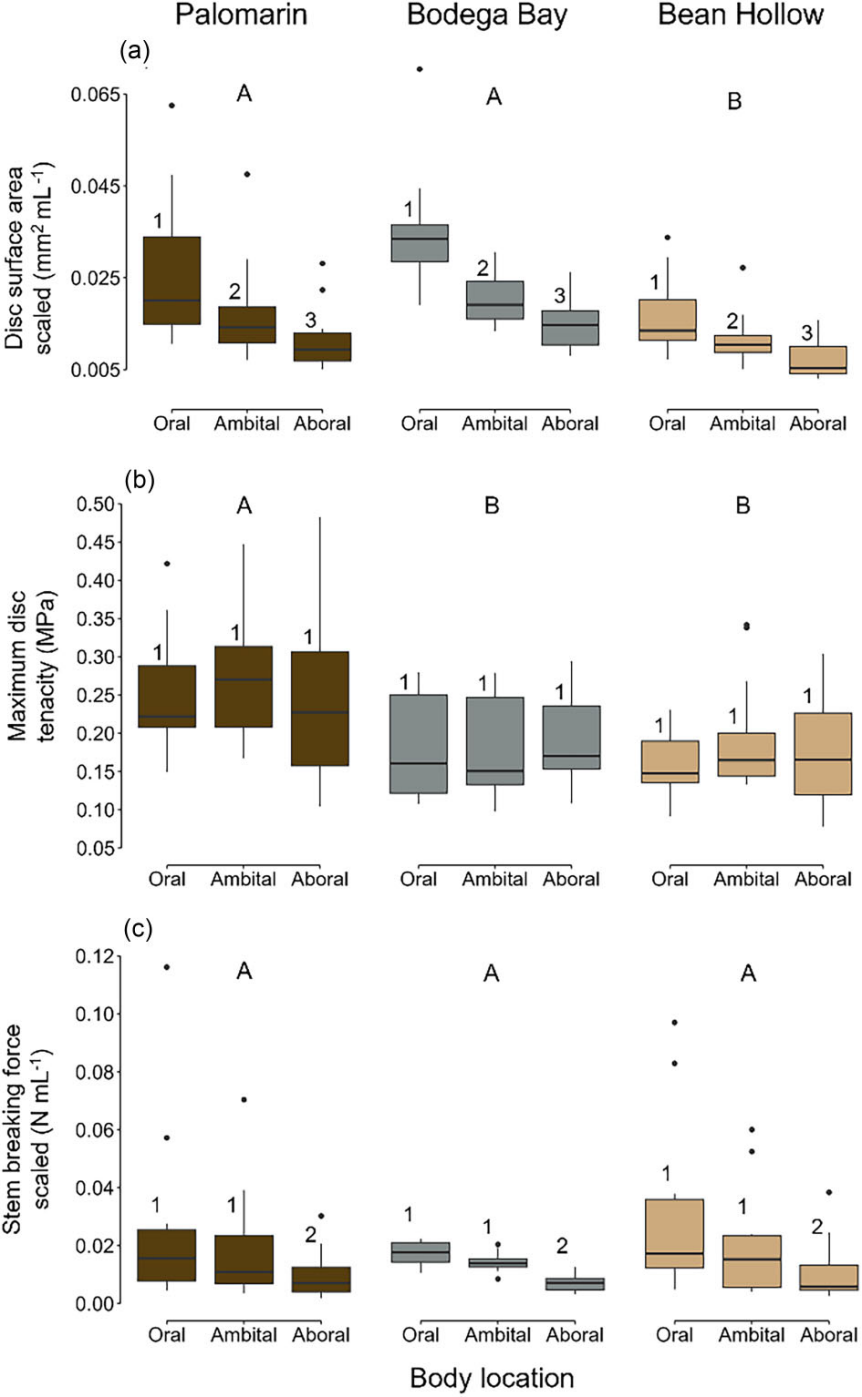
Tube foot disc surface area scaled by sea urchin volume (a; mm^2^ mL^−1^), maximum disc tenacity (b; MPa), and stem breaking force scaled by sea urchin volume (c; N mL^−1^), obtained from sea urchins collected from the three populations (*n* = 11–12 per population) that are color coded: Palomarin Beach (dark brown—mudstone native substrate), Bodega Bay (gray—native granite substrate), and Bean Hollow Beach (tan—native sandstone substrate). Disc surface area, maximum disc tenacity, and stem breaking force were measured on three body locations per sea urchin: oral (bottom section, facing the substrate), ambital (middle section), and aboral (top section, facing the water). The boxplot horizontal line is the median, and box edges are the 25th and 75th percentiles; whiskers indicate the largest value within 1.5 the interquartile distance (IQR) and points beyond are values greater than 1.5 the IQR but less than three times the IQR. Results from mixed linear models on disc surface area, maximum disc tenacity, and stem breaking force showed no significant interaction between population and body location, so Tukey post hoc analyses were conducted independently for population (shown as capital letters) and body locations (shown as numbers).

**Fig. 2:**
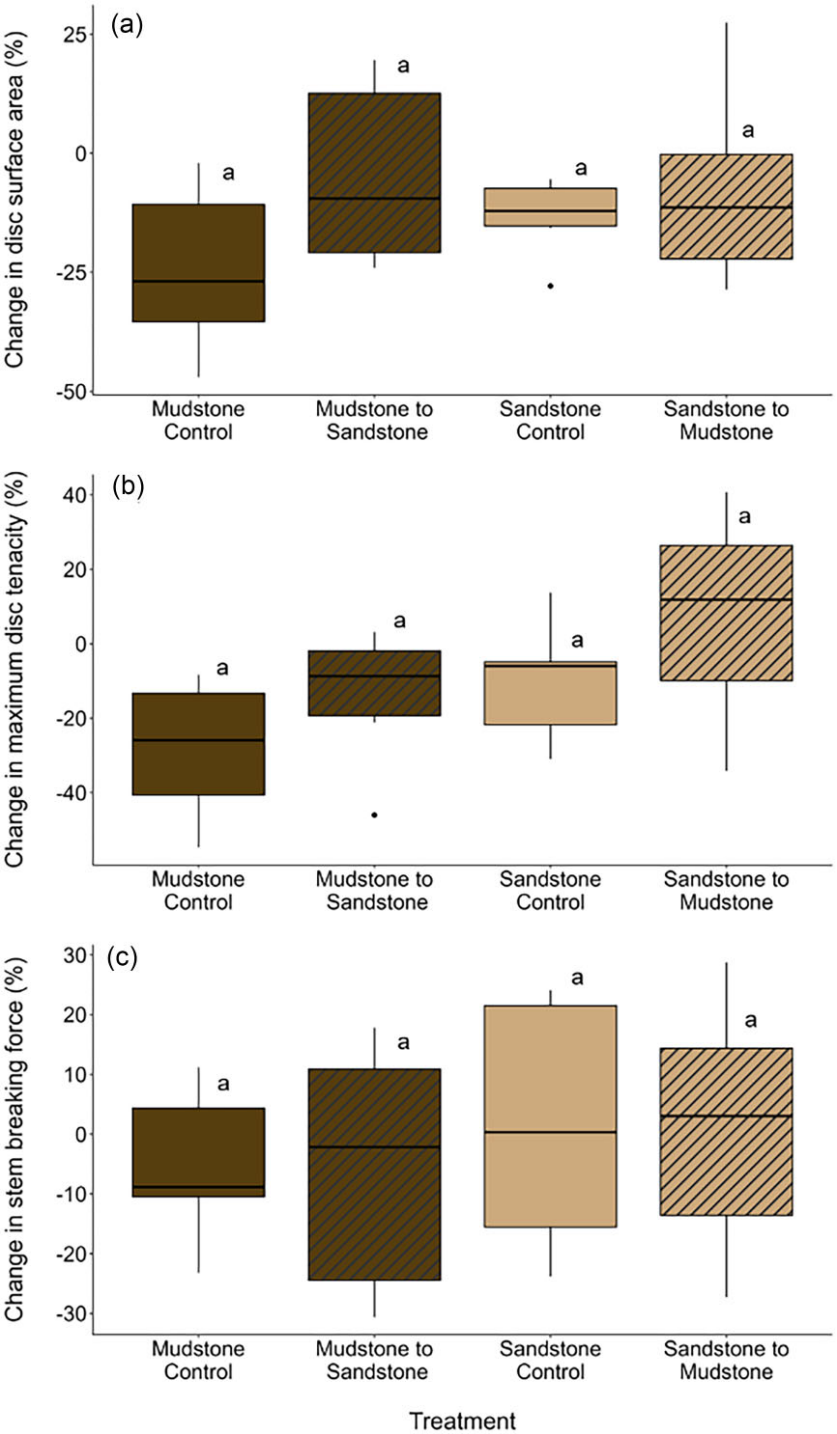
Percent change in tube foot disc surface area (a), maximum disc tenacity (b), and stem breaking force (c) of the laboratory-based reciprocal transplant experiment. Percent change was calculated as $( {\frac{{F - I}}{I}} ) \times \ 100$, where *I* represents the initial measurements obtained shortly after field collection (Day 0 or Day 14 in stem breaking force data) and *F* represents the final measurements at the end of the reciprocal transplant experiment (Day 109). The four treatments of the experiments were: Bean Hollow Beach urchins placed on their native substrate, sandstone (“sandstone control”—tan), Bean Hollow Beach urchins placed on mudstone substrate (“sandstone to mudstone”—tan striped), Palomarin Beach urchins placed on their native substrate mudstone (“mudstone control”—dark brown), and Palomarin Beach urchins placed on sandstone substrate (“mudstone to sandstone”—dark brown striped) (*n* = 6 per treatment). The boxplot horizontal line is the median, and box edges are the 25th and 75th percentiles; whiskers indicate the largest value within 1.5 the interquartile distance (IQR) and points beyond are values greater than 1.5 the IQR but less than three times the IQR. Substrate treatment had no effect on the response variables, so all treatments share the same letter.

Taking advantage of sea urchin plasticity over short timescales (Santos and Flammang 2007; Cohen-Rengifo et al. 2017; Narvaez et al. 2022), we conducted a reciprocal transplant experiment in the laboratory to assess whether the plasticity of tube foot morphology (disc surface area) and mechanical properties (maximum disc tenacity, stem breaking force) occur in response to sustained attachment to particular rock substrates. We used sea urchins from two populations: those native to mudstone (Palomarin Beach, *n* = 12) and those native to sandstone (Bean Hollow Beach, *n* = 12) because they showed the greatest differences in tube foot morphology (Figs. 1 and 2) as well as whole animal adhesive force (Stark et al. 2020). Sea urchins were shipped to Villanova University (Villanova, USA) within 72 h of collection. After arrival, sea urchins were placed on treatment substrates inside a 1000-L recirculating saltwater system.

Treatment substrates for individual sea urchin replicates were built with rocks taken from the sites where sea urchins were collected. Sandstone rocks were cut into 9 × 9 × 2-cm pieces with a wet masonry saw. Mudstone rocks shattered when cut, so flattened cobbles of similar dimensions to sandstone pieces were collected and sanded with 100-grit sandpaper to even the surface. Rock pieces were then embedded in 1.5–3 cm of marine epoxy (105; West Systems, Bay City, MI, USA) leaving 0.5–1 cm of the rock exposed above the epoxy. These substrate units had plastic mesh on the sides to isolate the individual and restrict sea urchin movement exclusively to the experimental substrate (see fig. S2 in Russell et al. 2018; Stark et al. 2020). Half of the sea urchins from each site were placed on their native substrate as a control and half were placed on a nonnative substrate (*n* = 6 per substrate treatment). Thus, sea urchins from the Palomarin Beach population were placed on their native substrate mudstone (“mudstone control”) and nonnative substrate sandstone (“mudstone to sandstone”) rock units, and sea urchins from the Bean Hollow Beach population were placed on their native substrate sandstone (“sandstone control”) and nonnative substrate mudstone (“sandstone to mudstone”) rock units. Sea urchins were fed rehydrated kelp (*Laminaria* sp. Wel Pac) *ad libitum*, feces were removed daily, and water temperature and salinity were monitored daily and maintained at 11 ± 0.8°C and 31 ± 0.5 PPT, respectively. Water chemistry (Ca, Mg, pH, P, and NH_3_) was monitored twice per week and corrected as needed.

Sea urchins used for this experiment were the same individuals collected for the field mensurative study used to assess disc surface area and maximum tenacity measurements (i.e., they experienced no previous destructive sampling). However, four animals from the population native to sandstone (Bean Hollow Beach) were replaced by extra sea urchins collected at that site on the same day because they showed signs of stress (losing spines). Disc surface area and maximum tenacity were measured in these four sea urchins 24 h after the others. Thus, disc surface area and maximum tenacity measured within 18–24 h of collection were considered “initial” values. For stem breaking force, “initial” values were measured 14 days after collection, once the animals were at Villanova University following the procedures described for the field mensurative study at BML. We waited 14 days to allow sea urchins to fully recover from the handling and shipping to not compound this stress with the stress associated with the destructive sampling required for stem breaking force measurement. Following the procedures described for the field mensurative study, tube foot disc surface area, maximum disc tenacity, and stem breaking force were measured on the oral side after 109 days of exposure to their treatment substrates. These measurements are termed “final” values. For each response variable, we calculated percent change (%) using the formula ${\mathrm{Percent\ change}} = ( {\frac{{F - I}}{I}} ) \times 100$, where *F* represents final values and *I* represents initial values.

#### Statistical analysis

Statistical analyses and graphs were executed in *R* (R Core Team 2022) using the package *nlme* (Pinheiro et al. 2018), and *ggplot2* (Wickham 2009).

In the field mensurative experiment, we used a linear mixed model to test for the fixed effects of population (Palomarin Beach: native to mudstone, Bodega Bay: native to granite, and Bean Hollow Beach: native to sandstone) and body location (oral, ambital, and aboral) on tube foot disc surface area scaled by sea urchin volume (mm^2^ mL^−1^), maximum disc tenacity (MPa), and stem breaking force scaled by sea urchin volume (N mL^−1^). We included sea urchin ID as a random factor in the model to account for multiple measurements on each individual. Data were transformed using ln when assumptions of normality of the model residuals (Shapiro–Wilk test) and homoscedasticity of the variance (Bartlett test) were not met (Table S1). Tukey post hoc comparisons for all analyses are available in the supplementary information (Table S3).

In the laboratory-based reciprocal transplant experiment, we used a one-way analysis of variance (ANOVA) to assess the effect of substrate treatment (“sandstone control,” “sandstone to mudstone,” “mudstone control,” and “mudstone to sandstone”) on tube feet disc surface area (mm^2^), maximum disc tenacity (MPa), and stem breaking force (N). A one-way ANOVA showed that sea urchin volume was comparable among treatments (*F*_3,44_ = 0.3062, *P* = 0.8508), so response variables were not scaled by sea urchin volume. Additionally, we used a one-sample, two-tailed *t*-test to assess whether percent change was significantly different from zero. A percent change significantly higher or lower than zero would show that the response variable significantly increased or decreased in value over the course of the experiment. Our results showed that substrate treatment did not affect percent change in any of the response variables (see the “Laboratory-based reciprocal transplant experiment” subsection in the “Results” section), so the data were combined for the *t*-test analyses. Assumptions of normality of the model residuals (Shapiro–Wilk test) and homoscedasticity of the variance (Bartlett test) were met for all response variables (Table S2).

## Results

### Mensurative field study

The mean and SE for all populations and body locations for each response variable (disc surface area, maximum disc tenacity, and stem breaking force) are available in the supplementary information (Table S4). The following sections detail the results of each morphological and mechanical property measured in the field.

#### Disc surface area

Tube foot disc surface area scaled by sea urchin volume (mm^2^ mL^−1^) was significantly affected by population and body location, but not their interaction (Table 1). Disc surface area, across all body locations, was larger in populations native to smoother substrates (Bodega Bay and Palomarin Beach) than those from a rougher substrate (Bean Hollow Beach) (Fig. 1a and Tables S3 and S4). Scaled disc surface area, across all populations, decreased along the oral–aboral axis, where tube feet on the oral body location had the largest discs, followed by tube feet on the ambitus, and then tube feet found on the aboral body location (Fig. 1a and Tables S3 and S4).

**Table 1:** Field mensurative experiment.

	Scaled disc area				Maximum disc tenacity				Scaled stem breaking force			
	Num DF	Den DF	*F*	*P*	Num DF	Den DF	*F*	*P*	Num DF	Den DF	*F*	*P*
Population	2	74	17.30	<0.001	2	74	12.06	<0.001	2	61	0.32	0.73
Body location	2	74	44.23	<0.001	2	74	0.77	0.47	2	61	11.79	<0.0001
Population: Body location	4	74	0.19	0.94	4	74	0.56	0.69	4	61	0.12	0.98

Analysis of variance table of the linear mixed model assessing sea urchin scaled tube foot disc area (mm^2^ mL^−1^), maximum disc tenacity (MPa), and scaled stem breaking force (mm^2^ mL^−1^) of sea urchins from three different populations. Fixed factors include population (native to mudstone [Palomarin Beach], native to granite [Bodega Bay], and native to sandstone [Bean Hollow Beach]) and body location (oral, ambital, and aboral). Sea urchin ID was included as a random factor to account for multiple measurements on the same individual. Num and den DF refer to the degrees of freedom associated with the model and model error, respectively.

#### Maximum disc tenacity

Maximum disc tenacity varied significantly among sea urchin populations, but not among body locations or their interaction (Table 1). Tube foot discs from the Palomarin Beach population, across all body locations, had higher maximum tenacity than discs from the Bean Hollow Beach population and Bodega Bay population (Fig. 1b and Tables S3 and S4). Maximum disc tenacity did not vary among body locations in any population (Fig. 1b and Table S4).

#### Stem breaking force

Stem breaking force scaled by sea urchin volume (N mL^−1^) varied significantly among body locations, but not among populations or the interaction between body location and population (Table 1). Stem breaking force, across all populations, decreased significantly along the oral–aboral axis. Tube feet on the oral side of the body required the highest force to break the stem, followed by tube feet on the ambitus, and then tube feet found on the aboral body location (Tables S3 and S4). Stem breaking force did not vary among populations in any body location (Fig. 1b, Table S4).

### Laboratory-based reciprocal transplant experiment

The mean and SE for the percent change of each response variable (disc surface area, maximum disc tenacity, and stem breaking force) are available in the supplementary information (Table S5). The following sections detail the results of the percent change for each morphological and mechanical property measured in the laboratory experiment.

#### Disc surface area

Substrate treatment did not alter the percent change of disc surface area (Table 2 and Fig. 2a). The *t*-test analysis, however, showed that when all treatments are combined, disc surface area percent change was significantly lower than zero (*t*_0.05,(2),23_ = −3.456, *P* = 0.002). This result shows that sea urchins significantly reduced their disc surface area during the experiment (mean percent change ± 95% CI = −12.52 ± 7.67%).

**Table 2:** Lab-based reciprocal transplant experiment.

		DF	SS	MS	*F*	*P*
Disc surface area	Substrate treatment	3	1356.60	452.20	1.52	0.24
	Residuals	20	5892.90	294.64		
Maximum disc tenacity	Substrate treatment	3	3869.80	1289.92	2.99	0.06
	Residuals	20	8635.8	431.79		
Stem breaking force	Substrate treatment	3	274.80	91.59	0.24	0.87
	Residuals	20	7629.00	381.45		

Analysis of variance table of the one-way ANOVA model assessing percent change in tube foot disc area, maximum disc tenacity, and stem breaking force from the laboratory-based reciprocal transplant experiment. The substrate treatments include “sandstone control,” “sandstone to mudstone,” “mudstone control,” and “mudstone to sandstone.”

#### Maximum disc tenacity

Percent change in maximum disc tenacity was not influenced by the substrate treatment (Table 2 and Fig. 2b). The *t*-test analysis showed that when all treatments are combined, percent change in maximum disc tenacity during the experiment was not significantly different from zero (*t*_0.05,(2),23_ = −2.050, *P* = 0.052; mean percent change ± 95% CI = −9.18 ± 9.95%).

#### Stem breaking force

Percent change in tube foot stem breaking force was not affected by the substrate treatment (Table 2 and Fig. 2c). The *t*-test analysis showed that when all treatments are combined, percent change in stem breaking force during the experiment was not significantly different from zero (*t*_0.05,(2),23_ = −2.050, *P* = 0.052; mean percent change ± 95% CI = −2.10 ± 3.63%).

## Discussion

The goal of this study was to determine whether the adhesive performance differences previously recorded among sea urchin populations of *S. purpuratus* (Stark et al. 2020) could be attributed to morphological or mechanical plasticity in the tube feet induced by adhering to substrates with different lithology in the field and in the laboratory. Specifically, Stark et al. (2020) found that populations native to mudstone and granite had higher adhesive performance than populations native to sandstone.

In our field mensurative study, we confirmed our first hypothesis and found tube foot morphological differences among populations. Namely, scaled tube foot disc surface area was larger in populations native to mudstone (Palomarin Beach) and granite (Bodega Bay) substrates than in the population native to the sandstone substrate (Bean Hollow Beach). Larger disc surface area results in higher disc attachment force (Narvaez et al. 2020) and may help explain the high whole animal attachment force and high total tube feet amputated under load in those populations reported previously (Stark et al. 2020). However, high whole animal attachment force may also be achieved by using more tube feet for attachment—a behavioral response (Cohen-Rengifo et al. 2019). Further studies assessing population-level behavioral differences in the number of tube feet used for adhesion are needed.

Several hypotheses, which are not mutually exclusive, may explain the differences in disc surface area observed in these populations. First, it is possible that these populations are genetically distinct, which leads to different disc surface area. However, the three study sites belong to the same biogeographic region (Blanchette et al. 2008), so they are likely part of the same metapopulation. Genetic studies on these population would help answer this question. Second, differential postsettlement selection that disproportionately affects sea urchins with smaller discs could explain the observed pattern. Indeed, smaller discs have lower attachment strength (Narvaez et al. 2020), which would facilitate dislodgement by predators and waves. However, sea urchins used in this study were collected from pits that decrease dislodgement risk by both reducing the effect of drag forces (Gaylord et al. 1994) and allowing the use of tube feet and spines (Jacinto and Cruz 2012). These studies suggest that deeper pits should offer more protection against wave dislodgement and predation than shallow pits. In our study, the populations native to the granite substrate (Bodega Bay) inhabit shallow and small pits, while the populations native to sandstone (Bean Hollow Beach) and mudstone substrates (Palomarin Beach) inhabit deeper and larger pits (Russell et al. 2018). Our results showed, however, that sea urchins in the populations native to the smooth substrates (i.e., granite, mudstone) had larger discs when compared to sea urchins from the population native to the rough sandstone substrate. Therefore, it is unlikely that the observed variation in tube foot disc surface area is related to postsettlement selection. Full assessment of the risk of dislodgment and predation for each of these populations is needed to determine this conclusively.

Observed differences in disc surface area may also be an environmentally induced plastic response driven by hydrodynamics, substrate lithology, or both. Previous studies have shown that tube foot discs regenerating in turbulent water movement regenerate larger discs than tube feet regenerating in quiescent conditions (Narvaez et al. 2022) and that *ex situ* conditions reduced adhesive performance (Toubarro et al. 2016; Cohen-Regifo et al. 2017). Although we do not have specific hydrodynamic information for the study sites, they are all open coast and have west–southwest exposure, suggesting overall similar hydrodynamic conditions (Narvaez et al. 2020). However, substrate lithology may explain why sea urchins native to mudstone and granite have larger disc area than sea urchins native to sandstone. In *P. lividus*, tube foot attachment strength on a smooth polymethyl-methacrylate (PMMA) substrate is lower than tube foot attachment strength measured on a rough PMMA. Because the viscoelastic tube foot disc can adapt to substrate surface rugosity, rough substrates effectively increase the attachment area of the disc when compared to the attachment area of smooth substrates (Santos et al. 2005). Thus, sea urchins native to mudstone and granite may have developed larger discs to cope with reduced attachment strength on smoother substrates.

Contrary to our second hypothesis, we found population differences in maximum disc tenacity, where sea urchins native to mudstone (Palomarin Beach) had higher maximum disc tenacity than sea urchins native to sandstone (Bean Hollow Beach) and granite (Bodega Bay). Substrate physical and chemical characteristics are known to affect sea urchin tenacity (Santos et al. 2009). For example, in *P. lividus*, disc tenacity is influenced by the surface energy of the substrate (Flammang 1996; Santos et al. 2005). In this experiment however, disc tenacity was measured on glass only, so any substrate-specific interaction with the adhesive secretion was removed. Tenacity is a mechanical property that is independent of disc surface area, so, when tested on a standard surface (i.e., glass capillary tube), changes in disc tenacity are likely due to changes in the adhesive secretion (e.g., amount and properties) which this study did not quantify. Moreover, previous studies have shown that sea urchins can use partial sections of the disc to adhere, creating incomplete adhesive contact (Santos et al. 2009; Stark et al. 2020). We, however, assumed that disc adhesive area and disc surface area were equivalent when attaching a single disc to the capillary tube, which may have overestimated the adhesive area. Future studies should include measurements of disc tenacity using true disc adhesive area (either full or partial contact) and compare the adhesive secretion composition and properties among these populations.

Our third hypothesis was supported, as stem breaking force was similar among the three populations, suggesting that fluctuating environmental conditions do not alter stem strength. Similarly, subpopulations of the sea urchin *P. lividus* found in sites with different hydrodynamic characteristics have similar stem breaking force, tensile strength, and stiffness, but the more exposed subpopulation had higher stem extensibility and toughness (Cohen-Rengifo et al. 2017). Future studies should incorporate more comprehensive stem mechanical property testing to explore differences in tube foot stems across populations.

Our last hypothesis predicted that adhesive performance would be higher in oral and ambital tube feet than aboral tube feet in sea urchins collected from deep pits (Palomarin Beach, Bean Hollow Beach), but sea urchins collected from shallow pits (Bodega Bay) would have similar ambital and aboral tube feet. Moreover, we expected that aboral tube feet would have equally poor performance across populations, as those tube feet are not often used to attach to the substrate and, thus, should not be influenced by substrate lithology. Our results, however, showed that disc surface area and stem breaking force increased along the aboral–oral axis in all populations, and that population differences in disc surface area occurred across all body locations. Together, these results suggest that the tube feet growth pattern dominates environmentally induced plasticity, as tube feet grow along the aboral–oral axis with oral tube feet being the oldest (Smith 1984). The mechanism used to strengthen the stem is unclear, but one possibility is that older tube feet have a thicker layer of mutable connective tissue, which is the tissue layer that bears the tensile load exerted on tube feet (Santos and Flammang 2005). Similar results were found in a closely related species, *S. droebachiensis*, where oral tube stems had higher breaking force and thicker stems than ambital and aboral stems (Leddy and Johnson 2000). Interestingly, maximum disc tenacity is similar among tube feet found in different body locations, suggesting that features of the adhesive secretion are maintained as sea urchins grow and tube feet move from the aboral to oral body location.

The goal of the laboratory-based reciprocal transplant experiment was to test the hypothesis that tube foot exposure to a nonnative substrate would induce tube foot morphological plasticity (disc surface area), but would not affect mechanical properties (maximum disc tenacity and stem breaking force). Contrary to our first hypothesis, but in support of the other two hypotheses, we found no effect of the substrate treatment on disc surface area, maximum disc tenacity, or stem breaking force. Instead, we detected an effect of time in the laboratory on disc surface area where, independent of the substrate treatment, discs were smaller by the end of the experiment. This plastic response of tube feet morphology to *ex situ* laboratory conditions suggests that environmental cues present in the natural environment influence tube foot morphology. In the laboratory, sea urchins were kept with minimal water turbulence, submerged, and fed *ad libitum*, but these conditions do not represent their natural intertidal habitat, where battering waves, changing tides, and food scarcity occur (Denny 1995). Decreased disc surface area in laboratory conditions may be a consequence of lower and less frequent hydrodynamic forces, which may act as a physical cue to increase attachment area (and thus attachment strength) in the field. Indeed, in environments with high hydrodynamic forces, there are fitness consequences to weak attachment strength (i.e., higher dislodgment and mortality), which are absent in laboratory conditions.

A decrease in tube feet adhesive performance after being transported to the laboratory (i.e., *ex situ*) has been previously reported in *S. purpuratus* (Narvaez et al. 2022). Quiescent laboratory conditions resulted in the regeneration of tube feet with smaller discs when compared to preamputation values and the shrinkage of nonamputated discs (Narvaez et al. 2020). Similarly, in *P. lividus*, the expression of the protein involved in disc adhesion (Toubarro et al. 2016), whole animal attachment force (Santos and Flammang 2006; Cohen-Rengifo et al. 2017, 2019), and stem mechanical properties (Cohen-Rengifo et al. 2019) decreased when animals were moved *ex situ*. These results, together with our findings, highlight an important, but often overlooked, aspect of phenotypic plasticity—potential plasticity in response to laboratory conditions. Future studies assessing the effect of substrate lithology on sea urchin adhesive performance should include a field-based reciprocal transplant that incorporates both substrate lithology and natural hydrodynamic conditions.

Phenotypic plasticity has been studied extensively because of its evolutionary and ecological importance (e.g., Pigliucci 1996; Agrawal 2001; Dewitt and Schneider 2005; Miner et al. 2005, Wennersten and Forsman 2012). In sea urchins, understanding the drivers of adhesive plasticity is needed to predict how population dynamics will be affected by the environment. This is particularly important in species like *S. purpuratus*, a key member of intertidal and subtidal communities as a bioeroder (Russell et al. 2018) and herbivore (Rogers-Bennett 2013). This study highlights how substrate lithology, laboratory conditions, and body location can alter *S. purpuratus* tube foot morphology and mechanical properties and influence adhesive performance.

## Supplementary Material

obae022_Supplemental_File

## Data Availability

Data are available in the supplemental information document.
