# Supplementary material for: Morphological and Mechanical Tube Feet Plasticity among Populations of Sea Urchin (Strongylocentrotus purpuratus)
Source: Integr Org Biol. 2024 Jul 8;6(1):obae022. doi: 10.1093/iob/obae022 (PMC11234643; doi:10.1093/iob/obae022)
Supplement: obae022_Supplemental_File [file obae022_supplemental_file.docx]

**Morphological and Mechanical Tube Feet Plasticity Among Populations of Sea Urchin (*Strongylocentrotus purpuratus)***

Carla A Narvaez^1,2^, Alyssa Y Stark^2^, Michael P Russell^2^

^1^ Department of Biology, Rhode Island College, 600 Mt. Pleasant Ave, Providence, Rhode Island 02908 USA

^2^ Department of Biology, Villanova University, 800 E. Lancaster Ave., Villanova, Pennsylvania 19085 USA

*author of correspondence: [cnarvaezdiaz@ric.edu](mailto:cnarvaezdiaz@ric.edu)

Running title: Tube feet plasticity among sea urchins populations

Table S1: Field mensurative study: Assumptions of normality and homoscedasticity of variances on disc area scaled by sea urchin volume, maximum disc tenacity, and stem breaking force scaled by sea urchin volume for the linear mixed model analyses.

| **Response variables** | **Factors** | **Transformation** | **Shapiro Wilk** | | **Bartlett Test** | |
| --- | --- | --- | --- | --- | --- | --- |
|  |  |  | W | p-value | K^2^ | p-value |
| Disc area | Population | ln | 0.990 | 0.619 | 2.505 | 0.286 |
|  | Body location |  |  |  | 1.028 | 0.598 |
| Max. disc tenacity | Population | ln | 0.988 | 0.0742 | 0.373 | 0.830 |
|  | Body location |  |  |  | 1.222 | 0.543 |
| Stem breaking force | Population | ln | 0.976 | 0.931 | 2.889 | 0.250 |
|  | Body location |  |  |  | 0.277 | 0.8705 |

Table S2: Lab-based translocation experiment: Assumptions of normality and homoscedasticity of variances on percent change of disc area, maximum disc tenacity, and stem breaking.

| **Response variables** | **Transformation** | **Shapiro Wilk** | | **Bartlett Test** | | |
| --- | --- | --- | --- | --- | --- | --- |
|  |  | W | p-value | Factors | K^2^ | p-value |
| Disc surface area | none | 0.9818 | 0.6564 | Population | 1.1201 | 0.2899 |
|  |  |  |  | Treatment | 1.0902 | 0.2964 |
| Max. disc tenacity | none | 0.9764 | 0.4374 | Population | 0.0145 | 0.9031 |
|  |  |  |  | Treatment | 0.1554 | 0.6934 |
| Stem breaking force | none | 0.9760 | 0.4261 | Population | 0.2628 | 0.6082 |
|  |  |  |  | Treatment | 0.9050 | 0.3414 |

Table S3: Field mensurative study: Tukey posthoc test for multiple comparisons of tube foot disc surface area scaled (mm^2^ mL^-1^), disc tenacity (MPa), and stem breaking force scaled (mm^2^ mL^-1^) of the linear mixed model.

| **Response** | **Level** | **Comparison** | **DF** | **Estimate** | **t ratio** | **P** |
| --- | --- | --- | --- | --- | --- | --- |
| Disc area | Body location | Aboral - Ambital | 75 | -0.419 | -4.702 | <0.0001 |
|  |  | Aboral - Oral | 75 | -0.837 | -9.407 | <0.0001 |
|  |  | Ambital - Oral | 75 | -0.419 | -4.705 | <0.0001 |
|  | Population | Bean Hollow-Bodega | 32 | -4.960 | -3.624 | 0.0002 |
|  |  | Bean Hollow-Palomarin | 32 | -0.3358 | -4.492 | 0.0001 |
|  |  | Bodega-Palomarin | 32 | 0.339 | 2.295 | 0.0787 |
| Max. disc tenacity | Population | Bean Hollow-Bodega | 32 | -0.0398 | -0.397 | 0.9173 |
|  |  | Bean Hollow-Palomarin | 32 | -0.3689 | -4.652 | <0.0001 |
|  |  | Bodega-Palomarin | 32 | -0.3292 | -3.282 | 0.0095 |
| Stem breaking force | Body location | Aboral - Ambital | 62 | -0.638 | -3.299 | 0.0045 |
|  |  | Aboral - Oral | 62 | -0.90716 | -4.736 | <0.0001 |
|  |  | Ambital - Oral | 62 | -0.278 | -1.437 | 0.3282 |

Table S4: Field mensurative study: Mean ± 1 SE for every location and population of tube foot disc surface area scaled (mm^2^ mL^-1^), maximum disc tenacity (MPa), and stem breaking force (mm^2^ mL^-1^)

|  |  | **Disc area** | | **Maximum disc tenacity** | | **Stem-breaking force** | |
| --- | --- | --- | --- | --- | --- | --- | --- |
| **Population** | **Body Location** | Mean | SE | Mean | SE | Mean | SE |
| Bean Hollow | Aboral | 0.0076 | 0.0013 | 0.1778 | 0.0217 | 0.0114 | 0.0037 |
| Bean Hollow | Ambital | 0.0116 | 0.0017 | 0.1961 | 0.0219 | 0.0210 | 0.0063 |
| Bean Hollow | Oral | 0.0168 | 0.0024 | 0.1590 | 0.0124 | 0.0323 | 0.0102 |
| Bodega Bay | Aboral | 0.0149 | 0.0017 | 0.1899 | 0.0171 | 0.0072 | 0.0010 |
| Bodega Bay | Ambital | 0.0207 | 0.0017 | 0.1804 | 0.0195 | 0.0143 | 0.0011 |
| Bodega Bay | Oral | 0.0353 | 0.0040 | 0.1803 | 0.0212 | 0.0173 | 0.0013 |
| Palomarin | Aboral | 0.0116 | 0.0020 | 0.2487 | 0.0362 | 0.0101 | 0.0029 |
| Palomarin | Ambital | 0.0175 | 0.0033 | 0.2720 | 0.0238 | 0.0199 | 0.0066 |
| Palomarin | Oral | 0.0261 | 0.0047 | 0.2534 | 0.0227 | 0.0277 | 0.0110 |

Table S5: Laboratory-based reciprocal transplant experiment: Mean ± 1 SE for the percent change (%) substrate treatments of tube foot disc surface area, maximum disc tenacity, and stem-breaking force.

|  | **Disc Area** | | **Maximum Disc Tenacity** | | **Stem breaking-force** | |
| --- | --- | --- | --- | --- | --- | --- |
| **Substrate Treatment** | Mean % change | SE | Mean % change | SE | Mean % change | SE |
| Mudstone Control | -24.41 | 5.06 | -28.25 | 5.33 | -5.33 | 3.73 |
| Mudstone to Sandstone | -4.71 | 5.65 | -13.80 | 5.23 | -5.64 | 6.07 |
| Sandstone Control | -13.27 | 2.37 | -10.11 | 4.73 | 1.45 | 6.27 |
| Sandstone to Mudstone | -7.71 | 5.93 | 7.41 | 8.11 | 1.10 | 6.09 |


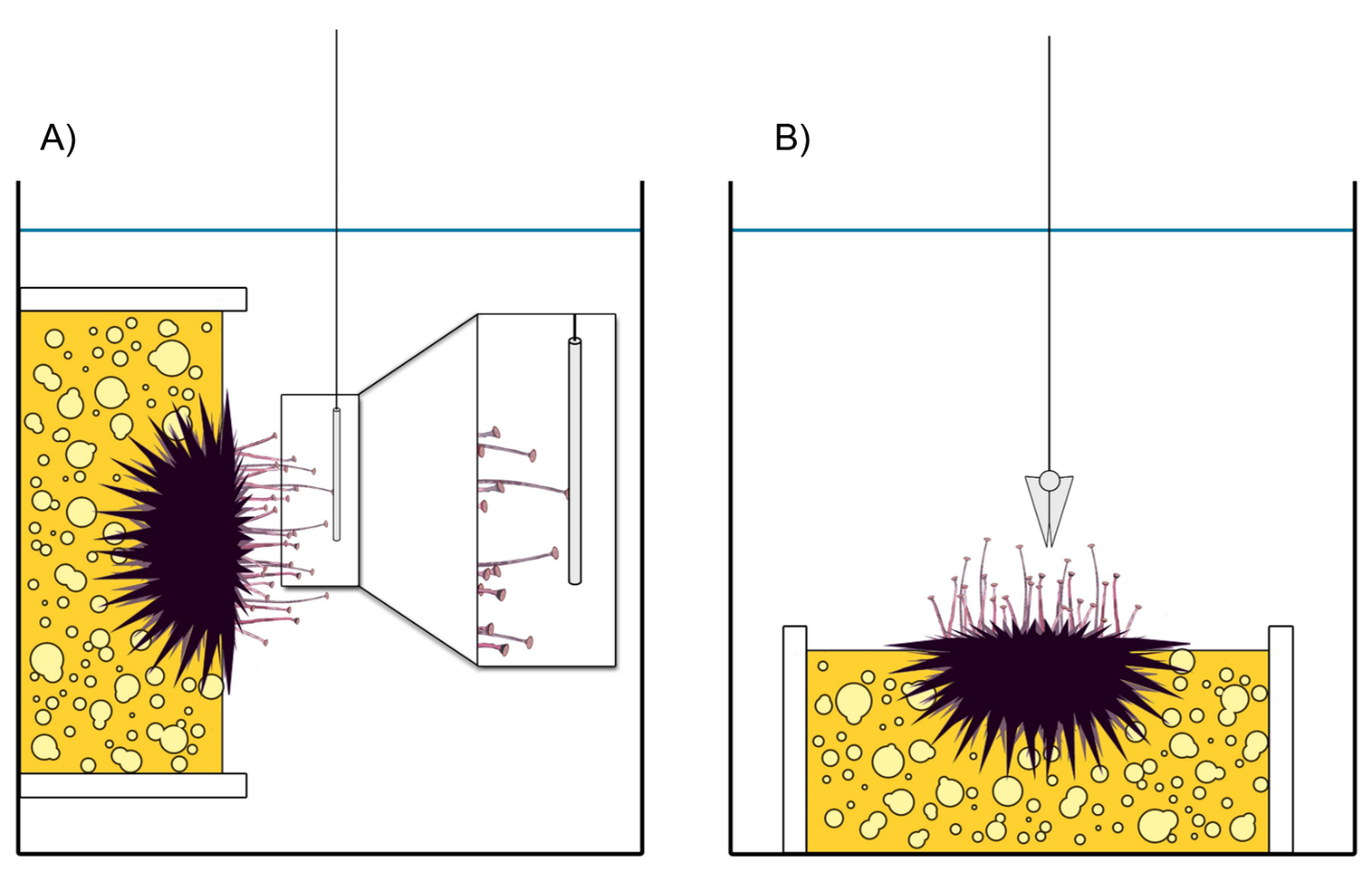


Fig S1: Schematic of the methodology used to collect the maximum disc attachment force (A) and stem breaking force (B) values. The sea urchin was restrained by a sponge (in yellow), exposing only the body location of interest, which in the diagram is the oral side. The sea urchin inside the sponge was then placed in a PVC tube (white bars holding the yellow sponge) that was submerged in sea water (blue line). The maximum attachment force of the disc (A) was assessed by attaching a capillary tube to a digital force gauge and, once a single tube foot was attached, a constant vertical force was applied and the force (N) required to detach was recorded. Stem breaking force (B) was obtained by attaching a metal clip to a digital force gauge, clipping the metal clip at approximately one half the length of the stem, and then a constant vertical force was applied and the force (N) required to break the stem was recorded. Image Credit: Andrew Moura
